# Ganglioside deficiency causes inflammation and neurodegeneration via the activation of complement system in the spinal cord

**DOI:** 10.1186/1742-2094-11-61

**Published:** 2014-03-28

**Authors:** Yuhsuke Ohmi, Yuki Ohkawa, Orie Tajima, Yasuo Sugiura, Keiko Furukawa, Koichi Furukawa

**Affiliations:** 1 Department of Biochemistry II, Nagoya University Graduate School of Medicine, 65 Tsurumai, Showa-ku, Nagoya 466-0065, Japan; 2 Department of Biomedical Sciences, Chubu University College of Life and Health Sciences, 1200 Matsumoto-cho, Kasugai 487-8501, Japan; 3 Department of Anatomy II, Nagoya University Graduate School of Medicine, 65 Tsurumai, Showa-ku, Nagoya 466-0065, Japan

## Abstract

**Background:**

Gangliosides, sialic acid-containing glycosphingolipids, are highly expressed in nervous systems of vertebrates and have been considered to be involved in the development, differentiation, and function of nervous tissues. Recent studies with gene-engineered animals have revealed that they play roles in the maintenance and repair of nervous tissues. In particular, knockout (KO) mice of various ganglioside synthase genes have exhibited progressive neurodegeneration with aging. However, neurological disorders and pathological changes in the spinal cord of these KO mice have not been reported to date. Therefore, we examined neurodegeneration in double knockout (DKO) mice of ganglioside GM2/GD2 synthase (B4GANLT1) and GD3 synthase (ST8SIA1) genes to clarify roles of gangliosides in the spinal cord.

**Methods:**

Motor neuron function was examined by gait analysis, and sensory function was analyzed by von Frey test. Pathological changes were analyzed by staining tissue sections with Klüver-Barrera staining and by immunohistochemistry with F4/80 and glial fibrillary acidic protein (GFAP). Gene expression profiles were examined by using DNA micro-array of RNAs from the spinal cord of mice. Triple knockout mice were generated by mating DKO and complement component 3 (C3)-KO mice. Gene expression of the complement system and cytokines was examined by reverse transcription-polymerase chain reaction (RT-PCR) as a function of age.

**Results:**

DKO mice showed progressive deterioration with aging. Correspondingly, they exhibited shrunk spinal cord, reduced thickness of spinal lamina II and III, and reduced neuronal numbers in spinal lamina IX, spinal lamina II, and spinal lamina I. Complement-related genes were upregulated in DKO spinal cord. Moreover, complement activation and inflammatory reactions were detected by GFAP-active astrocyte, microglial accumulation, and increased inflammatory cytokines such as tumor necrosis factor-alpha (TNFα) and interleukin-1-beta (IL-1β). Triple knockout mice showed restoration of reduced neuron numbers in the spinal cord of DKO mice, getting close to levels of wild-type mice.

**Conclusions:**

Disruption in the architecture of lipid rafts in the spinal cord was not so prominent, suggesting that mechanisms distinct from those reported might be involved in the complement activation in the spinal cord of DKO mice. Gene profiling revealed that inflammation and neurodegeneration in the spinal cord of DKO mice are, at least partly, dependent on complement activation.

## Background

Gangliosides, sialic acid-containing glycosphingolipids, are highly expressed in nervous systems of vertebrates [1]. Although gangliosides have been considered to be involved in the development, differentiation, and function of nervous tissues, recent studies with gene-engineered animals have revealed that they play roles mainly in the maintenance and repair of nervous tissues [2-4]. Almost all knockout (KO) mice disrupted of glycosyltransferase genes responsible for the synthesis of gangliosides exhibited neurodegeneration in the nervous systems [2]. Although GM2/GD2 synthase KO mice were reported first to show just subtle neurological dysfunctions at birth, progressive neurodegenerative changes were observed with aging [5-7]. GD3 synthase KO mice also exhibited reduced neuroregeneration of hypoglossal nerves after injury [8]. Compared with these KO mice of single genes, severe neurodegeneration with earlier onset and more intense pathological changes were detected in double KO of GM2/GD2 synthase and GD3 synthase genes (hereafter DKO) [9,10]. Audiogenic seizure was also observed in these mutant mice [11].

Although lack of all glycosphingolipids generated through glucosylceramide (GlcCer) resulted in embryonal lethality [12], conditional KO mice of GlcCer synthase, in which GlcCer synthase was disrupted in the brain after birth, also exhibited neurodegeneration [13]. Thus, lack of gangliosides might cause defects more or less in the maintenance of integrity of nervous systems, leading to neurodegeneration. However, neurodegeneration in the spinal cord caused by the lack of gangliosides have not been well investigated.

In some neurodegenerative diseases such as Alzheimer’s disease (AD) and Parkinson’s disease (PD), an important factor involved in the death of neurons is local inflammation [14], and the classic complement pathway was actually activated [15,16] in the brain tissues of AD patients. These results suggest that complement activation and subsequent inflammation are responsible for the degenerative changes in AD brain tissues, although the role of the complement system in AD is still controversial [17]. In many other neurodegenerative diseases, important roles of complement systems in the neuroinflammation and neurodegeneration have been also reported [18].

In this study, we examined abnormal neurological disorders and pathological changes in the spinal cord of DKO mice to confirm roles of inflammation in the induction of neurodegeneration in the spinal cord. Based on the DNA micro-array, we compared gene expression profiles between DKO and wild-type (WT) mice, and we found that complement-related genes were upregulated in DKO spinal cord. Therefore, we investigated whether complement activation brought about inflammatory reactions and finally neurodegeneration of the spinal cord in DKO mice. The DNA micro-array analysis combined with studies of gene-engineered animals resulted in the detection of profound findings in the pathogenesis and mechanisms for the neurodegeneration. Consequently, genetic approaches revealed that activation of the complement system is essentially involved in the inflammation and neurodegeneration in the spinal cord of DKO mice.

## Materials and methods

### Mice

The generation of KO mice of GM2/GD2 synthase [5] and GD3 synthase [8] was previously reported. To generate DKO mice efficiently, we also mated female homozygotes of the GD3 synthase gene and male heterozygotes of the GM2/GD2 synthase gene [9]. DKO mice with the two genes were designated as DKO (or GM3-only) mice, and wild-type mice for both genes were presented as WT mice. C3-KO mice (B6.129S4-*C3*^*tm1Crr*^/J) were obtained from The Jackson Laboratory (Bar Harbor, ME, USA). To generate triple KO (TKO) mice lacking GM2/GD2 synthase, GD3 synthase, and C3, DKO mice of GM2/GD2 synthase and GD3 synthase genes were mated with C3 KO mice, and genotypes of the offspring were screened for the three genes. Genotypes in C3-KO mice were screened as described [19]. WT and homozygous mutant mice of 4, 8, 15, 28 to 32, 42 to 50 weeks, and over 60 weeks after birth were used in this study. All experimental protocols were approved by the animal experimental committee of the Graduate School of Medicine in Nagoya University in accordance with the guidelines of Japanese government and were carried out in accordance with the National Institutes of Health Guide for the Care and Use of Laboratory Animals (1966). The number of animals used and their suffering were minimized.

### Antibodies

Antibodies used for Western immunoblotting were as follows: monoclonal anti-mouse C1q (rat IgG1) (Hycult Biotech, Uden, The Netherlands), monoclonal anti-β-actin (mouse IgG) (Sigma-Aldrich, St. Louis, MO, USA), or monoclonal anti-porcine glial fibrillary acidic protein (GFAP) (mouse IgG1) (Chemicon, Temecula, CA, USA). Chemiluminescence detection was performed by using horseradish peroxidase (HRP)-conjugated rabbit anti-rat IgG (H + L) (Zymed Laboratories, now part of Invitrogen, Carlsbad, CA, USA) and sheep anti-mouse IgG (Amersham Biosciences, now part of GE Healthcare, Little Chalfont, Buckinghamshire, UK). Antibodies used for immunohistochemistry were as follows: monoclonal anti-porcine GFAP (mouse IgG1) (Chemicon) and monoclonal anti-mouse F4/80 (rat IgG2b) (Serotec, Oxford, UK). Immunofluorescence detection was performed by using Alexa Fluor 488-goat anti-rat IgG2b (Invitrogen) or Alexa Fluor 555-goat anti-mouse IgG1 (Invitrogen).

### Primers

Primers used for real-time reverse transcription-polymerase chain reaction (RT-PCR) were designed according to Primer 3 Input™ [20] as shown in Additional file 1: Table S1.

### Extraction of glycolipids and thin-layer chromatography

Glycolipid extraction and thin-layer chromatography (TLC) were performed as described previously [21]. Briefly, lipids were extracted by chloroform/methanol at ratios of 2:1, 1:1, and then 1:2 sequentially. Glycolipids were isolated by a Florisil column after acetylation, and then neutral and acidic fractions were separated by DEAE-Sephadex (A-50) column chromatography.

### Footprint test

The footprint test is performed by applying Chromacryl (Chroma Acrylics, Lititz, PA, USA) to mouse feet. Usually, a red color is used on the forelimb and a blue color for the hind limb. Mice were then placed within a restricted cardboard tunnel (80 cm long × 10 cm wide × 10 cm high) with a white paper-covered floor, and footprints were made while the animal walked.

### von Frey test

Response to mechanical stimuli was examined by the von Frey test. To determine the paw withdrawal threshold to mechanical stimulation, the plantar surface of the hind paws in the WT and DKO mice was stimulated with a set of calibrated nylon monofilaments (von Frey hairs) with increasing force until the mice withdrew the limb. The minimum intensity of mechanical stimuli was taken as the force at which the mouse withdrew the paw.

### Klüver-Barrera staining

WT and DKO mice were perfused with phosphate-buffered saline (PBS) and then with 10% neutral buffered formalin (Wako, Osaka, Japan). Then the spinal cord was removed and was post-fixed with 10% neutral buffered formalin followed by Klüver-Barrera (KB) staining. The spinal cord was cut into lumber spinal cord segments L3-L4 and embedded in paraffin after dehydration and paraffin penetration. Blocked spinal cord was cut into 5 μm of cross-sections, and slices of spinal cord were stained with luxol fast blue solution and cresyl violet solution (KB staining). KB-stained slices of the spinal cord were observed by light microscopy.

### DNA microarray

The spinal cords isolated from 28- and 48-week-old mice (n = 3) were homogenized in Trizol™ (Invitrogen), and total RNA was extracted from tissues in accordance with the protocol of the manufacturer (Invitrogen). The quantity and purity of RNAs were checked by determining absorbance at 260/280 nm by using a spectrophotometer. Briefly, total RNA was reverse-transcribed to complementary DNA (cDNA) with T7-oligo (dT) primer (Affymetrix, Santa Clara, CA, USA). The cDNA synthesis product was used in an *in vitro* transcription reaction by using T7 RNA polymerase and biotinylated nucleotide analog (pseudouridine base). Then the labeled cRNA products were fragmented and loaded onto a CodeLink Uniset Mouse 20 K I Bioarray™ (Affymetrix) and hybridized in accordance with the protocol of the manufacturer. Streptavidin-phycoerythrin (Molecular Probes, now part of Invitrogen) was used as the fluorescent conjugate to detect hybridized target sequences. Signal intensity data from the GeneChip array were analyzed by GeneChip Operating Software (Affymetrix). Signal intensities for each spot were calculated by summation of the pixel intensities for each spot, and then the local background (based on the median pixel intensity of the area surrounding each spot) was subtracted. Array data normalization was performed independently for each slide by dividing each spot’s intensity (after background subtraction) by the median signal intensity of probes.

### Real-time reverse transcription-polymerase chain reaction

WT and DKO mice were perfused with PBS. The spinal cords were isolated from mice and homogenized in Trizol™. Total RNA was extracted from tissues by Trizol™ in accordance with the protocol of the manufacturer. After the quantity and purity of RNAs were checked by determining absorbance at 260/280 nm by using a spectrophotometer, total RNA was reverse-transcribed into cDNA by using M-MLV Reverse Transcriptase™ (Invitrogen) and oligo dT primer (Sigma-Aldrich). cDNA was frozen at −80°C until use.

Real-time RT-PCR was performed by using 4 ng cDNA per well, with F-400 or F-410 in a SYBR green qPCR kit™ (Finnzymes, Espoo, Finland) and a Thermal Cycler PTC-200™ (MJ Research, now part of Bio-Rad Laboratories, Inc., Hercules, CA, USA). The PCR conditions were as follows: preheating at 95°C for 10 minutes, 40 cycles of 95°C (10 seconds), 60°C (20 seconds), and 72°C (20 seconds). The plate reader was set at 78°C or 75°C (2 seconds) depending on individual primer pairs. For the internal control in the quantitative analysis, mGAPDH was used. Every sample was measured in duplicate, and the gene expression levels were analyzed by using Opticon Moniter3™ software (Bio-Rad Laboratories, Inc.).

### Western immunoblotting

The spinal cords were isolated after perfusion with PBS from mice and were homogenized in lysis buffer (10 mM Tris-HCl pH 7.4, 150 mM NaCl, 1 mM EDTA, 1 mM EGTA, 5 mM MgCl_2_, 50 mM NaF, 1 mM NaVO_4_, 1% Triton X-100, 200 mM PMSF, 0.01-0.02 TIU/mL aprotinin). The lysates were pelleted by centrifugation at 8,000 *g* for 60 minutes at 4°C. The supernatant was centrifuged at 18,000 *g* for 90 minutes at 4°C, and clarified lysates were used for immunoblotting. Lysates were frozen at −80°C until use.

Lysates from the spinal cord of WT and DKO mice were mixed with SDS sample buffer, heated at 100°C for 5 minutes, and resolved by a 10% to approximately 12% SDS-PAGE. Proteins were transferred onto polyvinylidene difluoride membranes by semi-dry electrophoresis for 60 minutes at 15 V. Membranes were incubated in PBST (PBS containing 0.1% Tween 20) with 5% skim milk for 2 hours at room temperature or overnight at 4°C, then incubated with primary antibodies for 1 hour at room temperature or overnight at 4°C, followed by HRP-conjugated second antibodies for 45 minutes at room temperature. Bands were visualized by ECL™ (Western Lightning Chemiluminescence Reagent; PerkinElmer Life Sciences, Waltham, MA, USA).

### Immunohistostaining

WT and DKO mice were perfused with PBS and then 4% paraformaldehyde in 0.1 M phosphate buffer. The spinal cord was removed, post-fixed with 4% paraformaldehyde in 0.1 M phosphate buffer overnight at 4°C, then replaced with 10% sucrose for 6 hours, 15% sucrose for 6 hours, 20% sucrose for 6 hours, and embedded in OCT compound (Sakura Finetechnical, Nagoya, Japan) and frozen in liquid nitrogen. Frozen sections were set at longitudinal orientation for the spinal cord and were cut into 7 μm-thick sections on a cryostat (CM3050S; Leica, Wetzlar, Germany) at −20°C. The sections were placed on MAS-coat grids (Matsunami Glass, Osaka, Japan), and tissue slices were stored at −80°C until used.

For immunohistochemistry, tissue slices were washed twice with PBS for 5 minutes at room temperature, blocked with 10% normal goat serum for 1 hour at room temperature, and incubated with primary antibodies for 1 hour at room temperature or overnight at 4°C. Then slices were washed 3 times with PBS for 5 minutes at room temperature, and incubated with Alexa Fluor-conjugated second antibodies for 45 minutes at room temperature. Then they were washed 3 times with PBS for 5 minutes at room temperature, and were sealed with PermaFlour™ aqueous mounting medium (Thermo Fisher Scientific Inc., Waltham, MA, USA). Cover slips were mounted and observed by fluorescence microscopy.

### Enzyme-linked immunosorbent assay

After perfusion with PBS, the spinal cords were isolated from mice and homogenized and sonicated in lysis buffer (10 mM Tris-HCl pH 7.4, 150 mM NaCl, 1 mM EDTA, 1 mM EGTA, 5 mM MgCl_2_, 50 mM NaF, 1 mM NaVO_4_, 1% Triton X-100, 1 mM PMSF, 0.01-0.02 TIU/mL aprotinin) (spinal cord 100 mg tissue/300 μL lysis buffer). The lysates were centrifuged at 8,000 *g* for 60 minutes at 4°C, and the supernatants were centrifuged at 18,000 *g* for 90 minutes at 4°C. Five hundred μg/50 μL of tissue lysates was used for the assay. Levels of interleukin-1-alpha (IL-1α), IL-1β, and tumor necrosis factor-alpha (TNFα) proteins were determined by using assay kits for mouse IL-1α/IL1F1™, mouse IL-1β/IL-1 F2™ enzyme-linked immunosorbent assay (ELISA), or mouse TNFα/TNFF1A™ (R&D Systems, Minneapolis, MN, USA). The assay was performed in accordance with the instructions of the manufacturer.

### Statistical analysis

All results were presented as the mean ± standard deviation and were initially subjected to the Bartlett, Hartley, and Levene test for homogeneity of variance. The Shapiro-Wilk test was used to verify that the data followed a normal distribution. Statistical significance was calculated by using Student *t* test in cases of two comparison groups and one-way analysis of variance (ANOVA) followed by Tukey-Kramer *post hoc* test in cases of more than two comparison groups. Two-way ANOVA followed by Bonferroni *post hoc* test was used comparing mouse group and time course. All statistical significances were set at *P <0.05, **P <0.01, ***P <0.001.

## Results

### Abnormal phenotypes of mice by deletion of GM2/GD2 synthase and GD3 synthase

**Figure 1:**
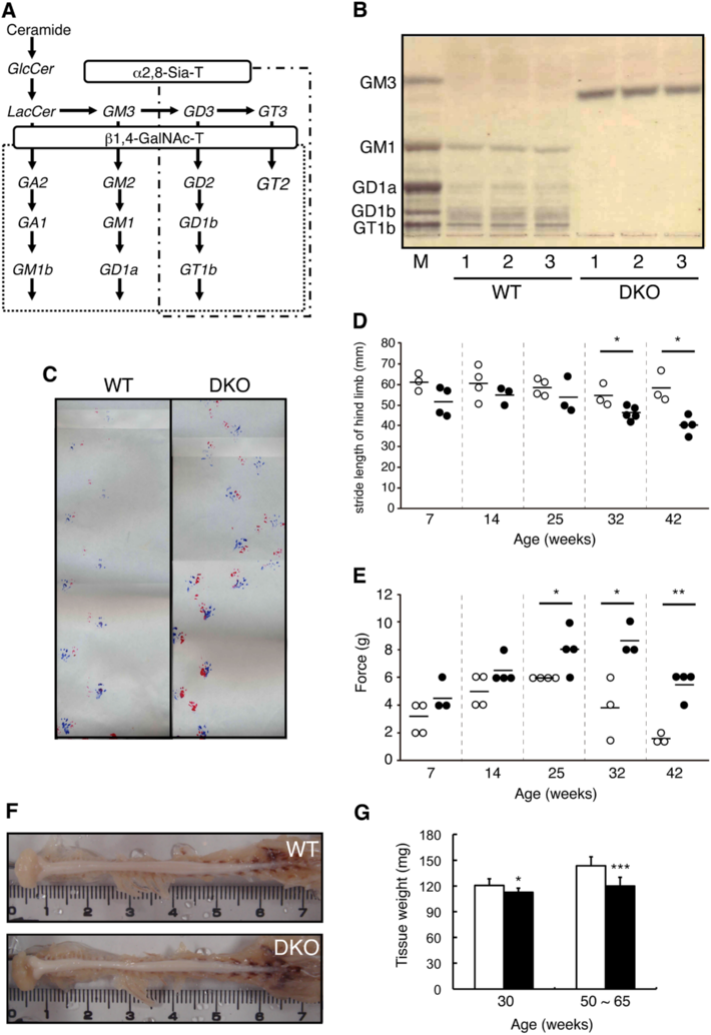
**Abnormal phenotype in double knockout (DKO) mice. (A)** A synthetic pathway of gangliosides. Structures boxed in two squares were depleted in DKO mice. **(B)** Thin-layer chromatography (TLC) of gangliosides from spinal cord of 15-week-old wild-type (WT) and DKO mice was performed with control gangliosides from bovine brain (lane M). Resorcinol was used for the detection. **(C)** Gait pattern was examined by the footprint test in 70- to 80-week-old mice. A red color was used for the forelimb and a blue color for the hind limb. **(D)** Average values for stride length of hind limb in individual age WT and DKO mice. The number of tissues examined was n = 3 or 4 (WT, open circles; DKO, closed circles). Data were analyzed by two-way analysis of variance (ANOVA). Comparison of WT and DKO with same age revealed that they were statistically significant by Bonferroni *post hoc* test (shown in figure, *P <0.05; **P <0.01). **(E)** von Frey strings test in individual age WT and DKO mice. The number of tissues examined was n = 3 or 4 (WT, open circles; DKO, closed circles). Two-way ANOVA analysis of data was performed as described in **(D)**. Comparison of WT and DKO with same age was done, and the results are shown (*P <0.05; **P <0.01). **(F)** Photographs of fixed spinal cord from 44-week-old WT and DKO mice. Scale: centimeter (cm). **(G)** Weights of the spinal cord of WT and DKO mice. The numbers of tissues examined were n = 11~13 (WT, open columns; DKO, closed columns). Data were presented as mean ± standard deviation (SD) and were analyzed by Student *t* test (*P <0.05; ***P <0.001).

We generated DKO mice lacking the GM2/GD2 synthase and the GD3 synthase genes (Figure 1A) and investigated phenotypes of the DKO mice. Ganglioside composition in the spinal cord as analyzed by TLC exhibited only GM3 in DKO mice (Figure 1B). In the footprint test, DKO mice walked in a zigzag line (Figure 1C) and with abnormal stride (Figure 1D) whereas WT mice showed straight walking. These abnormal gates were exacerbated with aging in DKO mice (Figure 1D). Interaction of mouse group and time course did not turn out to be statistically significant (*P* = 0.2624). The differences among time courses in DKO mice (but not WT mice) turned out to be statistically significant (WT, *P* = 0.674; DKO, *P* = 0.0167) (Figure 1D). Sensory nerve functions in the 7-, 14-, 25-, 32-, and 42-week-old mice were examined with the von Frey test. Sensitivity to mechanical pain stimuli in the hind limbs apparently reduced at 25 weeks after birth or later (Figure 1E). Interaction of mouse groups and time courses showed *P* = 0.0311. The results of comparison among time courses in mice were as follows: WT, *P* = 0.00226; DKO, *P* = 0.00173 (Figure 1E). These results suggested that abnormal nerve functions occurred because of the lack of gangliosides in DKO mice. Furthermore, the spinal cords of 44-week-old DKO mice were thinner than those in WT mice (Figure 1F) and weighed less in the DKO mice both at 30 weeks (WT: 120.6 ± 7.8 mg, n = 11 versus DKO: 112.6 ± 4.9 mg, n = 10) and at 50 to 65 weeks after birth (WT: 143.6. ± 10.5 mg, n = 13 versus DKO: 120.0 ± 10.1 mg, n = 12) (Figure 1G).

### Neurodegeneration of the spinal cord in double knockout mice

**Figure 2:**
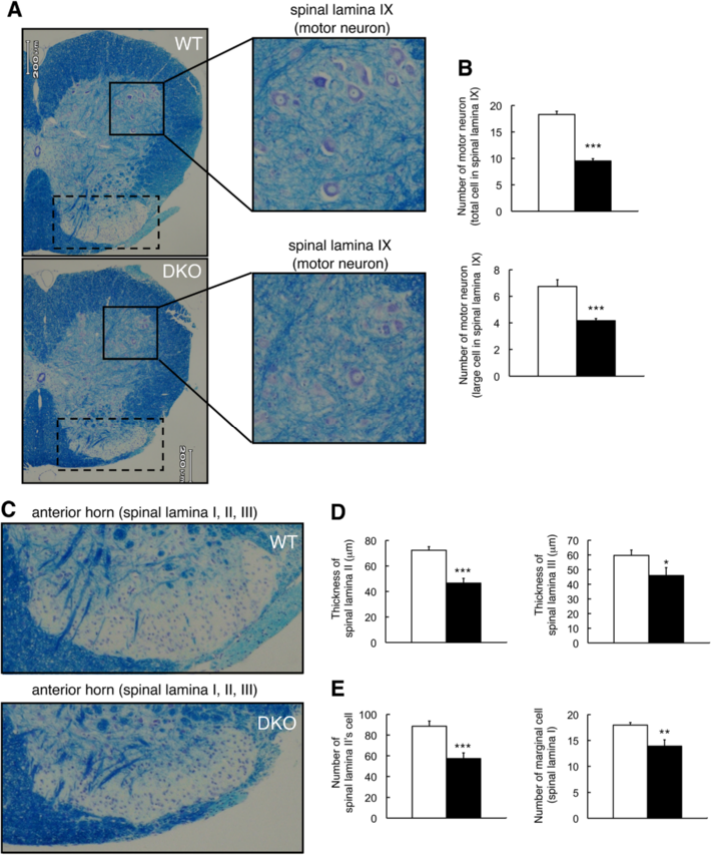
**Neurodegeneration in the spinal cord of double knockout (DKO) mice. (A)** Spinal cord sections of 44-week-old mice were stained with Klüver-Barrera (KB). Enlarged images indicated spinal lamina XI (motor neuronal region) in the spinal cord as shown by square. **(B)** The numbers of motor neuron in the spinal lamina XI of lumber spinal cord segments L3 were counted and presented as the number of motor neurons. **(C)** An enlarged anterior horn—spinal lamina I, II, and III indicated by dashed squares in **(A)**—of spinal cord sections stained with KB. **(D)** Thicknesses of spinal lamina II and III were measured in lumber spinal cord segments L3 in wild-type (WT) and DKO mice and presented as the thicknesses of spinal lamina II and III. **(E)** The numbers of spinal lamina II neurons and marginal cells in spinal lamina I were counted and presented as the number of neurons. The numbers of mice examined are as follows: 44-week-old WT (n = 4; open columns) and DKO (n = 4; closed columns); data are presented as mean ± standard deviation (SD) and were analyzed by Student *t* test (*P <0.05; **P <0.01; ***P <0.001). The thickness of sections was 5 μm.

To examine whether histopathological changes related to abnormal gait and reduced sensitivity to mechanical pain stimuli occur in the spinal cord of DKO mice, we performed KB staining for lumber spinal cord 3 (L3) of spinal cord from 44-week-old WT and DKO mice (Figure 2A). There was an apparent loss of motor neurons in the DKO mice as shown in spinal lamina IX (Figure 2A and B). The thickness of spinal lamina II and III decreased in DKO mice compared with WT mice (Figure 2C and 2D). The number of neuronal cells in spinal lamina II and that of marginal cells (in spinal lamina I) also decreased in DKO mice compared with WT mice (Figure 2D). These results suggested that abnormal phenotypes found in gait and sensitivity to mechanical pain stimuli were caused by neurodegeneration in the spinal cord of DKO mice.

### Genes of complement components were upregulated in the spinal cords of double knockout mice

**Figure 3:**
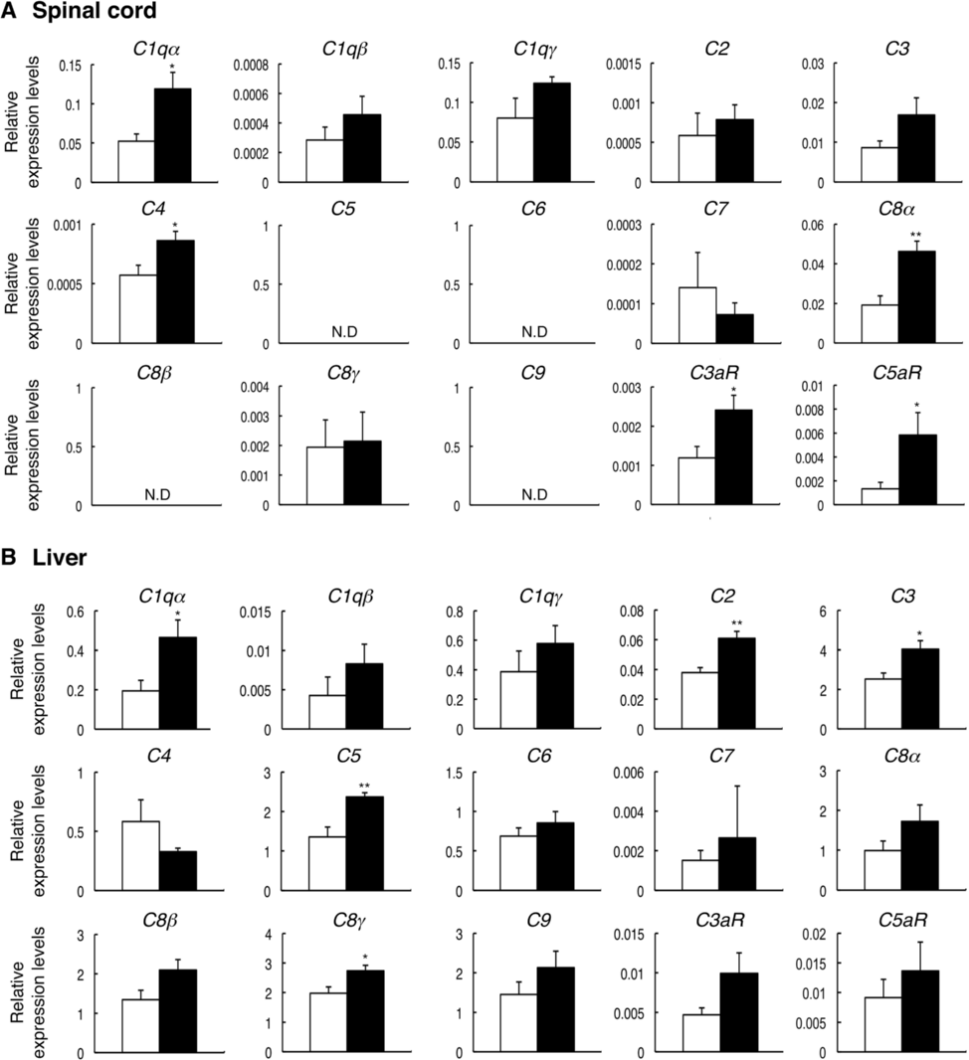
**Expression levels of complement components were upregulated in the spinal cord and liver of wild-type (WT) and double knockout (DKO) mice.** mRNA levels of complement genes (*C1qα*, *C1qβ*, *C1qγ*, *C2*, *C3*, *C4*, *C5*, *C6*, *C7*, *C8α*, *C8β*, *C8γ*, *C9*, *C3aR*, and *C5aR*) in the spinal cord **(A)** and liver **(B)** of WT and DKO mice were analyzed by real-time reverse transcription-polymerase chain reaction (RT-PCR) and presented after correction with the mouse *GAPDH* gene. The numbers of mice examined were as follows: 28-week-old male WT (n = 3; open columns) and DKO (n = 3; closed columns); data were presented as mean ± standard deviation (SD) and were analyzed by Student *t* test (*P <0.05; **P <0.01). ND, not detected.

To investigate the mechanisms by which neurodegeneration was caused in DKO mice, we performed a DNA microarray analysis with mRNAs from the spinal cords of 28- and 48-week-old mice. Among genes differentially expressed between WT and DKO mice, complement genes (*C1qα*, *C4*, and *C3aR*) were found to be generally upregulated in the spinal cord (Table 1). The expression level of *C4* was markedly upregulated in both the 28- and 48-week-old DKO mice (30-week-old WT: 4.07 versus DKO: 10.27 and 48-week-old WT: 6.71.07 versus DKO: 14.09). The expression level of *C3aR*, receptor for anaphylatoxin C3a, was most strongly upregulated in the 48-week-old DKO mice (WT: 0.29 versus DKO: 1.44). To precisely determine the gene expression profiles, we performed real-time reverse transcription-PCR (RT-PCR) with mRNAs from individual mice. Consequently, the expression level of *C4* was upregulated in DKO mice compared with WT at all ages examined (Additional file 1: Figure S1A). The expression level of *C3aR* was upregulated in all DKO male mice and many DKO female mice (Additional file 1: Figure S1B). These results indicated that *C4* and *C3aR* were upregulated regardless of the presence of skin injury and more definitely in male DKO mice than in female. Since complement system functions in innate immunity and inflammatory reactions with sequential activation of diverse components, we performed real-time RT-PCR for complement system-related genes by using mRNAs from the spinal cord and liver of 28-week-old mice. The expression levels of *C1qα*, *C4*, *C5*, *C3aR*, and *C5aR* were significantly upregulated in the spinal cord of DKO mice (Figure 3A). The expression levels of *C1qβ*, *C1qγ*, and *C3* in the spinal cord tended to increase in the DKO mice. The expression of *C6*, *C7*, *C8β*, and *C9* could not be detected. These results suggested that the majority of complement-related genes were upregulated in the spinal cord of DKO mice. On the other hand, the expression levels of complement-related genes were generally higher in the liver than in the spinal cord, and *C4* was persistently low in the liver of DKO mice compared with WT mice (Figure 3B). Thus, the expression of these genes in the central nervous system (CNS) seemed to be regulated in a distinct manner from that in the liver (Additional file 1: Figure S2).

**Table 1:** Relative expression levels of complement components in the spinal cord using DNA microarray

**Weeks**	**Gene**	**WT (expression)**	**DKO (expression)**	**Ratio**
	*C1qα*	9.84	15.79	1.60
28 weeks	*C4*	4.07	10.27	2.53
	*C3aR*	0.34	0.51	1.52
	*C1qα*	11.17	24.14	2.16
48 weeks	*C4*	6.71	14.09	2.10
	*C3aR*	0.29	1.44	5.04

Expression levels of genes in wild-type (WT) and double knockout (DKO) mice were quantified based on normalized signal intensities (see Materials and methods) obtained from DNA microarray.

### Complement components were chronologically upregulated in the spinal cord of double knockout mice

**Figure 4:**
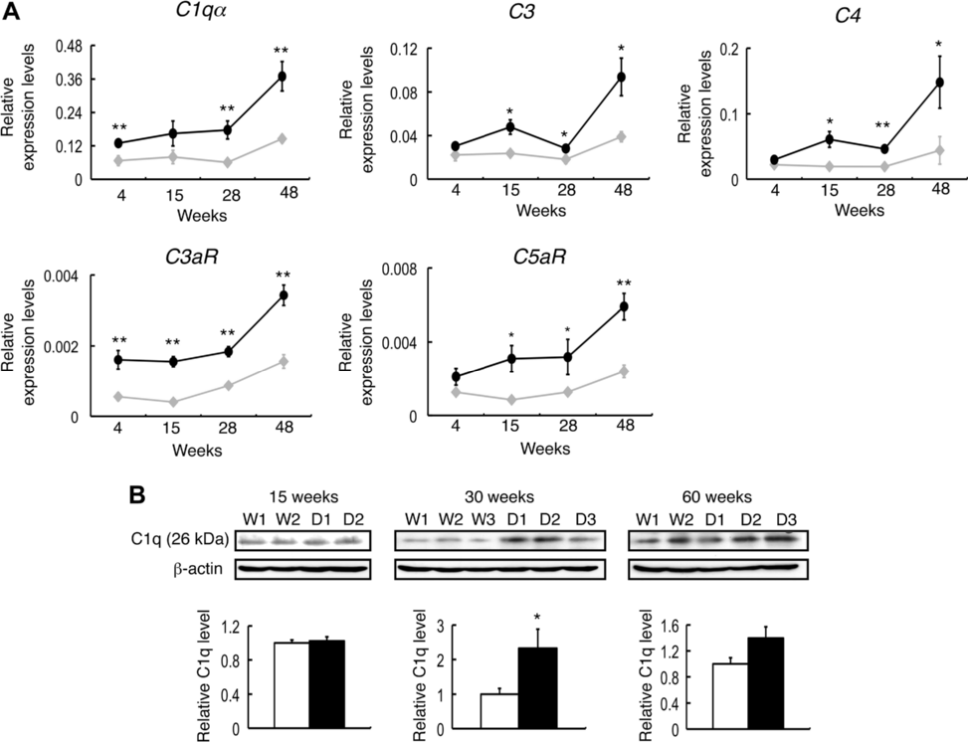
**Complements were upregulated with aging in the spinal cord of double knockout (DKO) mice. (A)** mRNAs from spinal cord of 4-, 15-, 28-, and 48-week-old wild-type (WT) and DKO mice were analyzed for the expression levels of complement genes with real-time reverse transcription-polymerase chain reaction (RT-PCR) and presented after correction by mouse *GAPDH* gene (gray diamond, WT; closed circle, DKO). The numbers of mice examined were as follows: 4-, 15-, 28-, and 48-week-old WT (n = 3) and DKO (n = 3); data are presented as mean ± standard deviation (SD), and analysis of data by two-way analysis of variance (ANOVA) was performed as described in Figure 1D. Interaction of mouse groups and time courses: *C1qα*, *C3*, and *C4*, *P* <0.01; *C3aR* and *C5aR*, *P* <0.05. The comparison among time courses in mice revealed that all genes in WT and DKO mice, except for *C4* in WT mice, turned out to be statistically significant. Comparison of WT and DKO with same age is shown in figure (*P <0.05; **P <0.01) **(B)** Western immunoblotting of C1q in the spinal cord was performed by using total lysates from 15-, 30-, and 60-week-old WT and DKO mice. The intensity of the bands was corrected with that of β-actin and presented as mean ± SD. The numbers of mice examined were as follows: 15-week-old WT (n = 2), DKO (n = 2); 30-week-old WT (n = 3), DKO (n = 3); 60-week-old WT (n = 2), DKO (n = 3); data are presented as mean ± SD and were analyzed by Student *t* test (*P <0.05; **P <0.01).

Because the phenotypes of the DKO mice were exacerbated with aging, we performed real-time RT-PCR to examine chronological changes in the expression levels of *C1qα*, *C3*, *C4*, *C3aR*, and *C5aR* by using mRNAs from the spinal cord of 4-, 15-, 28-, and 48-week-old WT and DKO mice (Figure 4A). All of these genes showed no or minimal differences at 4 weeks after birth between WT and DKO mice. The expression levels of complement genes were then upregulated in DKO mice with aging. These results suggested that upregulation of complement genes took place along with neurodegeneration in the nervous tissues of DKO mice. Furthermore, we performed immunoblotting to investigate the protein levels of C1q in the spinal cord of the DKO mice. Since C1q proteins are essential in the complement system, we examined the C1q protein in the spinal cord of 15-week-old mice, showing equivalent levels between WT and DKO mice. But it increased 2.3-fold in 30-week-old DKO mice and just tended to be higher in 60-week-old DKO mice (Figure 4B), whereas its mRNAs were upregulated with aging. There results suggested that complement components were consumed with complement activation.

### Abnormal proliferation of astrocytes and accumulation of microglia in the spinal cord of double knockout mice

**Figure 5:**
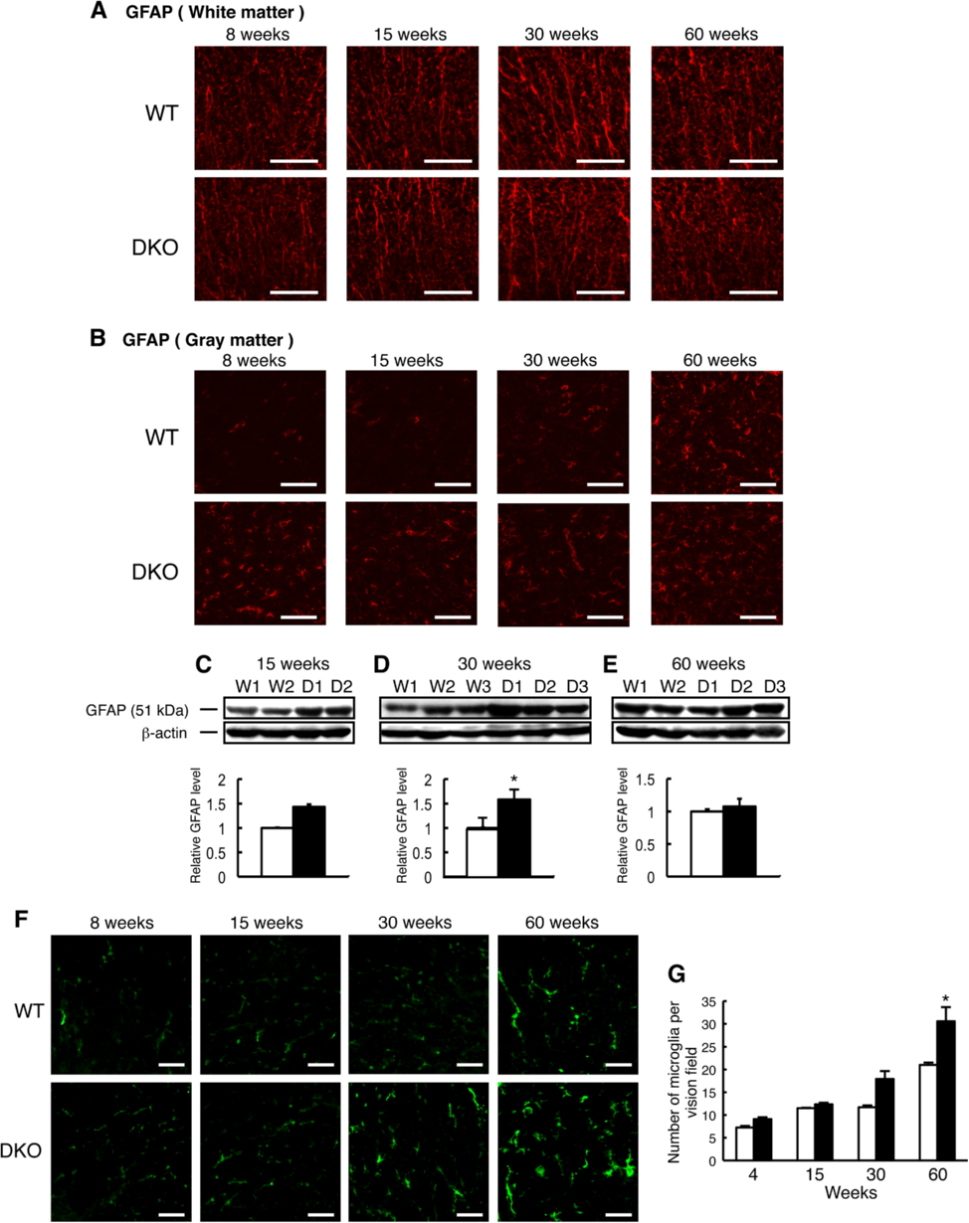
**Immunohistostaining of the spinal cord for** glial fibrillary acidic protein **(GFAP) and F4/80. (A, B)** Immunohistostaining of GFAP in the spinal cord of 8-, 15-, 30-, and 60-week-old wild-type (WT) and double knockout (DKO) mice. GFAP (astrocyte marker) was detected with monoclonal anti-GFAP and Alexa Fluor 555-conjugated anti-mouse IgG1. Staining patterns of GFAP showed no difference in the white matter between WT and DKO mice at all stages examined. In the gray matter, GFAP staining in 8- and 15-week-old DKO mice increased. Eventually, there was no difference between the WT and DKO mice at 30 and 60 weeks. **(C-E)** Western immunoblotting of the spinal cord lysates from 15-, 30-, and 60-week-old WT and DKO mice. GFAP increased in the 15-week-old DKO mice compared with WT mice, but the difference in GFAP protein levels between WT and DKO mice reduced with aging. Beta-actin levels were also analyzed to confirm equal sample loading. The numbers of mice examined were as follows: 15-week-old WT (n = 2), DKO (n = 2); 30-week-old WT (n = 3), DKO (n = 3); 60-week-old WT (n = 2), DKO (n = 3); data are presented as mean ± standard deviation (SD). **(F)** Immunohistostaining of F4/80 (microglia marker) in the spinal cord of 8-, 15-, 30-, and 60-week-old WT and DKO mice by using monoclonal anti-mouse F4/80 and Alexa Fluor 488-conjugated anti-rat IgG2b. **(G)** The number of microglia was quantified as an average in the visual field of the fluorescence microscope (×400). Microglia numbers significantly increased in 60-week-old DKO mice compared with the WT mice. The numbers of mice examined were as follows: 4-, 15-, and 30-week-old WT (n = 2), DKO (n = 2), 60-week-old WT (n = 3), DKO (n = 3); data are presented as mean ± SD. Thickness of sections was 10 μm (scale bar, 50 μm). Data were analyzed by Student *t* test (*P <0.05).

To investigate whether inflammation occurs in the spinal cord of DKO mice, we performed immunohistostaining of astrocytes (GFAP) and microglia (F4/80). We found no definite difference in GFAP staining of white matter between WT and DKO mice at 8 to about 60 weeks after birth (Figure 5A). On the other hand, GFAP-active astrocytes in gray matter was found more prominently in 8-, 15-, and 30-week-old DKO mice than in WT mice (Figure 5B), whereas no difference was found between WT and DKO mice in 60-week-old mice. The increased GFAP expression in DKO mice with aging was confirmed by immunoblotting (Figure 5C-E). GFAP bands increased at 15 to about 30 weeks after birth in DKO mice compared with WT mice but were almost equivalent at 60 weeks after birth between WT and DKO mice. Accumulation of microglia tended to increase at 30 weeks after birth and significantly accumulated hereafter, whereas no difference could be found at 8 to approximately 15 weeks after birth between DKO and WT mice (Figure 5F and G).

### Secretion of inflammatory cytokines in the spinal cord of double knockout mice

**Figure 6:**
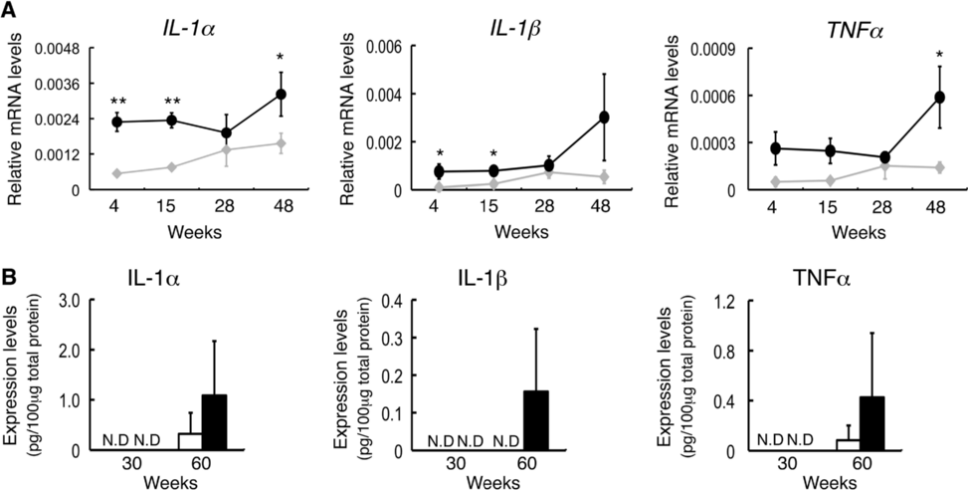
**Inflammatory cytokine levels increased in the spinal cord of double knockout (DKO) mice with aging. (A)** mRNAs from the spinal cord of individual mice were analyzed for the expression levels of cytokine genes with real-time reverse transcription-polymerase chain reaction (RT-PCR) and corrected by the mouse *GAPDH* gene. Compared with wild-type (WT) mice, expression levels of interleukin-1-alpha (*IL-1α*), *IL-1β*, and tumor necrosis factor-alpha (*TNFα*) in the spinal cord of the DKO mice were upregulated with aging (diamond, WT; circle, DKO). The numbers of mice examined were as follows: 4-, 15-, 28-, and 48-week-old WT (n = 3) and DKO (n = 3). Analysis of data by two-way analysis of variance (ANOVA) was performed as described in Figure 1D. For interaction of mouse groups and time courses, all genes were not statistically significant. In the comparison among time courses in mice, only *TNFα* in DKO mice turned out to be statistically significant (*P* = 0.0424). Comparison of WT and DKO with same age is shown in figure (*P <0.05; **P <0.01). **(B)** Protein levels of IL-1α, IL-1β, and TNFα in the spinal cord of 30- and 60-week-old WT and DKO mice were analyzed with enzyme-linked immunosorbent assay (ELISA). The numbers of mice examined were as follows: 30- and 60-week-old WT (n = 3) and DKO (n = 3); data are presented as mean ± standard deviation (SD) and were analyzed by Student *t* test (*P <0.05). ND, not detectable.

To investigate whether abnormal proliferation of astrocytes and accumulation of microglia are associated with actual inflammation, we measured mRNA expression levels of inflammatory cytokines (*IL-1α*, *IL-1β*, and *TNFα*) in the spinal cord of WT and DKO mice with real-time RT-PCR. Expression levels of *IL-1α* and *IL-1β* were significantly upregulated in 4- and 15-week-old DKO mice, and those of *IL-1α* and *TNFα* were significantly upregulated in 48-week-old DKO mice (Figure 6A). Expression levels of *IL-6* and *INFγ* could not be detected in either type of mice. We then performed ELISA for the inflammatory cytokines. None of these inflammatory cytokines was detected at 30 weeks after birth, but they tended to increase at 60 weeks (Figure 6B). These results suggested that the spinal cord of DKO mice underwent inflammatory reaction, and it was exacerbated with aging.

### Alleviation of inflammation and neurodegeneration in the spinal cord of double knockout mice by deletion of complement C3

**Figure 7:**
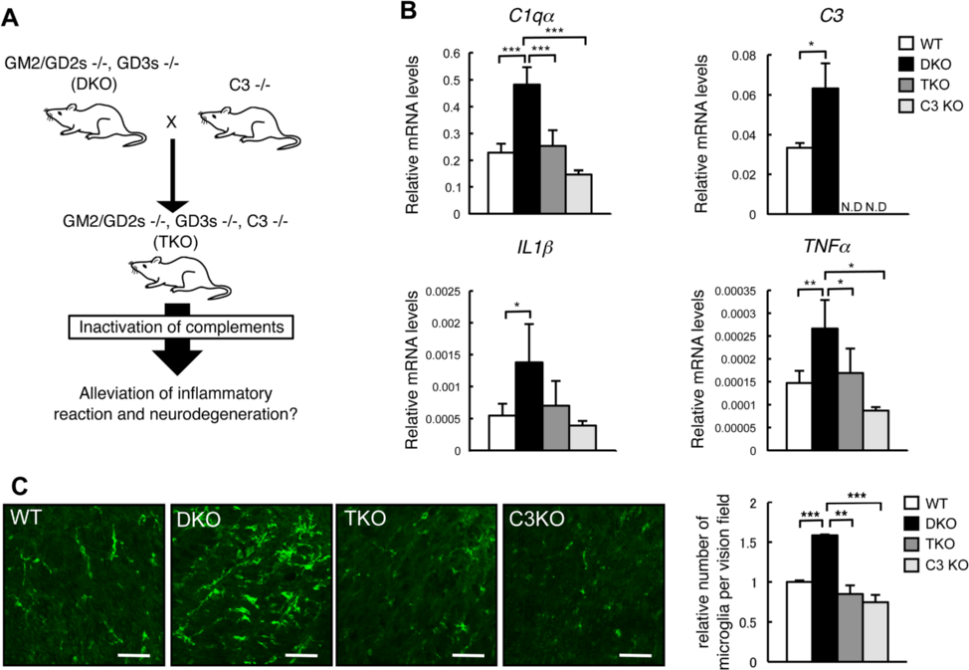
**Alleviation of the complement activation, inflammation, and neurodegeneration in double knockout (DKO) mice by disruption of complement C3 gene. (A)** To clarify the involvement of complement systems in the inflammatory reactions and neurodegenerations in DKO mice, DKO- and C3-deficient mutant mice were mated, generating triple knockout (TKO) mice. **(B)** Expression levels of complement mRNAs from the cerebellum of 40-week-old mice were analyzed for *C1qα*, *C3*, *IL-1β*, and *TNFα* genes with real-time reverse transcription-polymerase chain reaction (RT-PCR). Relative expression levels are presented after correction by the *GAPDH* gene. The numbers of mice examined were as follows: wild-type (WT) (n = 5), DKO (n = 5), TKO (n = 5), and C3KO (n = 3); data are presented as mean ± standard deviation (SD) and were analyzed by one-way analysis of variance (ANOVA) with Tukey-Kramer *post hoc* test (*P <0.05; **P <0.01; ***P <0.001). **(C)** Immunohistostaining of F4/80 (microglia marker) in the spinal cord of 40-week-old WT and DKO mice by using monoclonal anti-mouse F4/80 and Alexa Fluor 488-conjugated anti-rat IgG2b. The number of microglia was quantified as an average in a visual field of the fluorescence microscope (×400). Numbers of section area examined were as follows: WT (n = 3), DKO (n = 3), TKO (n = 3), and C3KO (n = 3); data are presented as mean ± SD and were analyzed by one-way ANOVA with Tukey-Kramer *post hoc* test (**P <0.01; ***P <0.001). The thickness of sections was 7 μm (scale bar, 50 μm).

To investigate whether the inflammatory reaction and neurodegeneration in DKO mice are due to complement activation and can be rescued by disruption of complement systems, we generated TKO mice by mating the DKO mice of GM2/GD2 synthase and GD3 synthase genes with C3 KO mice (Figure 7A). As expected, expression levels of *C3* were null in the TKO mice (Figure 7B), and *C1qα* in TKO also reduced to levels almost equivalent to that in WT mice. By measuring mRNA expression levels of inflammatory cytokines (*IL-1β* and *TNFα*), expression of *TNFα* was downregulated in the TKO mice, whereas *IL-1β* tended to decrease when compared with that in DKO mice (Figure 7B). As for F4/80 immunohistostaining, accumulation of microglia completely became equivalent between TKO and WT mice (Figure 7C). These results indicated that inflammatory reactions observed in DKO mice almost subsided in TKO mice.

**Figure 8:**
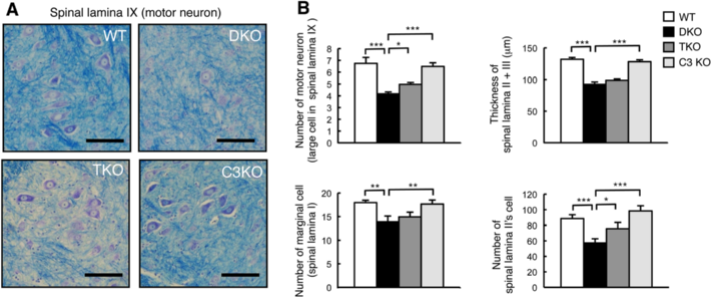
**Restoration of reduced neuron numbers in double knockout (DKO) mice by disruption of complement C3 gene. (A)** Spinal cord sections of 44- to 50-week-old mice stained with Klüver-Barrera (KB). Enlarged images indicated XI layer (motor neuronal region) in the spinal cord. **(B)** The numbers of motor neurons were counted in the spinal lamina XI of lumber spinal cord segments L3 and presented as the number of motor neurons. The thickness of sections was 5 μm (scale bar, 100 μm). The thickness of spinal lamina II + III was measured in lumber spinal cord segments L3 in wild-type (WT) and DKO mice and presented as the thickness of spinal lamina II + III. The numbers of spinal lamina II neurons and marginal cells in spinal lamina I were counted and presented as the number of neurons. The numbers of mice examined were as follows: 44- to 50-week-old WT (n = 4), DKO (n = 4), triple knockout (TKO) (n = 5), and C3KO (n = 3); data are presented as mean ± standard deviation (SD) and were analyzed by one-way analysis of variance (ANOVA) with Tukey-Kramer *post hoc* test (*P <0.05; **P <0.01; ***P <0.001).

To clarify whether the neurodegeneration of the spinal cord in DKO mice could be restored by disruption of complement systems, L3 of the spinal cord in 44- to 50-week-old mice was stained by KB staining. As described in Figure 2, there was a clear loss of neurons in DKO mice compared with WT mice. The number of motor neurons (spinal lamina IX cells) and neuronal cells in the spinal lamina II was fairly restored in TKO mice, but thicknesses of spinal lamina II + III and marginal cells (in spinal lamina I) were almost equivalent between DKO and TKO mice (Figure 8A and B). These results suggested that the inflammatory reaction and neurodegeneration observed in DKO mice were definitely alleviated in the TKO mice. However, neurodegeneration in the spinal cord could not be completely restored in the TKO mice compared with the WT mice.

## Discussion

Aberrant expression of gangliosides have been reported in a variety of neurological disorders and their animal models (for example, in the spinal cord of amyotrophic lateral sclerosis (ALS) [22], brain tissues of patients with ALS [23], and brains of patients with PD [24]). These results suggested that ganglioside administration might bring about beneficial effects in these diseases. In particular, GM1 was reported to be effective for the treatment of PD in primates [25], and randomized controlled studies for patients with PD have been tried, showing that it is safe and may provide some clinical benefit to the patients [26]. GM1 has been also used for the treatment of spinal cord injury [27]. These results suggested that gangliosides are deeply involved in the maintenance of the integrity of nerve tissues, and supplement of them may be a choice as therapeutic agents for the neurodegenerative diseases.

In this study, the relationship between neurological disorders and pathological damages in the spinal cord of DKO mice was examined. Motor neuron deficits as shown in abnormal gait and sensory nerve disorders as demonstrated in elevated threshold to the pain stimulation of von Frey test deteriorated with aging. Correspondingly with these behavioral abnormalities, sizes in the spinal cord and spaces of individual compartments such as dorsal horn and ventral horn shrank as shown in Figure 2. All of these findings suggested that complex gangliosides are essential for the maintenance of the integrity of the spinal cord as shown in various knockout mice of ganglioside synthase genes [2,3,28].

As for human diseases that are modeled by ganglioside-deficient mice, “infantile-onset symptomatic epilepsy syndrome” can be raised [29]. The responsible gene for this disease turned out to be GM3 synthase. More directly, seven cases of “complex hereditary spastic paraplegia” with GM2/GD2 synthase mutations (eight mutations) were reported [30]. In these cases, similar abnormal features with our KO mice could be seen, suggesting usefulness of the KO mice for the analysis of these diseases.

The gene expression profiling using RNA from the spinal cord of 28- and 48-week-old WT and DKO mice revealed that complement systems in DKO were generally activated. Not only main components of the complement system but also their receptor genes were also upregulated, suggesting increased consumption of them. Recently, involvement of inflammation and immune systems in the neurodegeneration has been increasingly recognized [31], and roles of complement systems in the physiological and pathological processes in CNS have been extensively studied [18,32,33]. Although the majority of components in the complement system are expressed in the brain tissues [32,34], no studies on complements in the spinal cord have been reported so far.

In various neurodegenerative diseases such as AD, an important factor involved in the death of neurons is local inflammation [14,35]. All components involved in the classic complement pathway could be found in neurons and glial cells [34], and this pathway was activated as shown in fibrillar β-amyloid [15] or neurofibrillary tangles [16] in the brain tissues of patients with AD. These results strongly suggested that complement activation and subsequent inflammation are responsible factors for the degenerative changes in AD brain tissues but that the role of the complement system in AD pathogenesis and progression is complex [17]. Indeed, absence of C1q leads to less neuropathology in mouse models of AD [36]. Furthermore, treatment with a C5aR antagonist decreases pathology and enhances behavioral performance in AD model mice [37]. In many neurodegenerative diseases other than AD, including traumatic damages, important roles of complement systems in the neuroinflammation and neurodegeneration have been also reported [18]. In ALS, complement is significantly involved in the disease model [38] and its inhibitor might be beneficial for the treatment of ALS [39].

As expected, TKO mice generated by mating DKO mice of two ganglioside synthases and C3 gene KO mice showed apparent improvement in the inflammatory reaction, microgliosis, and neurodegeneration as shown in the sizes and neuron numbers in the spinal cord. Although the restoration of these degenerative features was not complete (that is, not equivalent to that of WT mice), they showed significant improvement, suggesting that inflammatory reaction via complement activation should be a main mechanism for the neurodegeneration due to ganglioside deficiency.

How complement systems are activated by the lack of gangliosides is not known at this time. Recently, innate immune responses within CNS have been widely recognized as playing a major role in the development of autoimmune disorders and neurodegeneration such as multiple sclerosis and AD [40]. Among neuro-immune regulatory proteins (NIReg), GPI-anchored proteins, molecules of the immunoglobulin superfamily (like siglecs), and complement C3a and factor H are included. In particular, complement regulatory factors such as H and I were reported to bind to gangliosides, resulting in the inactivation of alternative pathway of complement [41]. They demonstrated the ability of sialic acid on glycolipids to promote factor H binding to C3b, resulting in the suppression of the alternative activation pathway. It was also reported that sialic acid on a microbial surface bound defined site of factor H, resulting in the increased conversion of bound C3b to iC3b [42]. This might be a mechanism for serum resistance of *Neisseria gonorrhoae*. In these cases, inhibitory effects of gangliosides were dose-dependent and not specific for the site of sialic acid substitution in gangliosides [41].

In addition, patients with hemolytic uremic syndrome due to factor H mutation were actually reported [43,44]. These results suggest that complement activation in DKO mice might come, at least partly, from insufficient inhibition of complement activation with factor H due to loss of gangliosides. Correspondingly, erythrocytes from DKO mice lacking complex gangliosides showed increased sensitivity to complement (rabbit)-dependent hemolysis (unpublished data). On the other hand, a report also indicates that gangliosides activate complement system by using an *in vitro* system [45].

In turn, gangliosides have been reported to play roles in the regulation of functions and architecture of membrane microdomains in the neural tissues [28,46]. In our study of cerebellum in DKO mice, disrupted lipid rafts were demonstrated [47,48]. However, fractionation of membrane extracts with Triton X-100 revealed that the distribution of raft markers was not so disturbed in the spinal cord of DKO mice. Therefore, the mechanisms for the neurodegeneration due to ganglioside deficiency might not necessarily be the same between cerebellum and spinal cord.

As shown above, even TKO mice did not achieve complete restoration of abnormal findings indicating inflammation and neurodegeneration. In particular, astrocytosis in the nerve tissues did not decrease by the genetic deletion of C3, suggesting not only activation of the complement system but involvement of some other factors in the inflammatory reaction in the spinal cord of DKO mice. Among them, disturbed regulation of various membrane molecules on neurons and glial cells due to ganglioside deficiency should be included. In particular, functional disorders in the receptors such as nerotrophic receptors (TrkB) [49], ion channels [50], NMDA receptor [51], and muscarinic acetylcholine receptors [52] should be considered. Activation of proteases such as calpain might also be involved as reported [53]. We are now conducting trials to identify those factors.

Recently, it has been reported that the complement system may play beneficial roles in the development and maintenance of the nervous systems [54,55]. Genetic inactivation of the complement system resulted in the various abnormal features in the nervous systems; for example, failure of anatomical refinement of retinogeniculate connections and the retention of excess retinal innervation by lateral geniculate neurons were observed in C1q-deficient mice [54]. Reduced clearance of fibrillar amyloid-beta by microglia was found in C3 and Mac-1 KO mice [56]. Furthermore, complement receptor 2^−/−^ mice exhibited prominent increases in basal neurogenesis [57]. In our results, C3-deficient mutant mice showed slightly higher neuron numbers in spinal lamina II, suggesting that sufficient clearance of deteriorating neuronal tissues might not be achieved during the development in C3-deficient mice.

As previously described, the complement system plays important roles in both neuroprotection and neurodegeneration [25] and seems to be a dual-edged sword [18]. The fact that TKO mice lacking a complement system showed apparently improved histology in the spinal cord compared with DKO mice suggests that influences of complement deficiency in the development of mice were not so serious as neuroinflammation and neurodegeneration, which exacerbated with aging (that is, in the maintenance of nerve tissues after birth). Mechanisms for complement activation due to ganglioside deficiency in the spinal cord remain to be clarified.

Disruption in the architecture of lipid rafts in the spinal cord was not so prominent as in brain tissues, suggesting that mechanisms distinct from those reported might be involved in the complement activation in the spinal cord of DKO mice. Gene expression profiling revealed that inflammation and neurodegeneration in the spinal cord of DKO mice are dependent, at least partly, on complement activation due to ganglioside deficiency.

## Abbreviations

AD: Alzheimer’s disease; ANOVA: analysis of variance; C3: complement component 3; CNS: central nervous system; DKO: double knockout; ELISA: enzyme-linked immunosorbent assay; GFAP: glial fibrillary acidic protein; GlcCer: glucosylceramide; HRP: horseradish peroxidase; IL: interleukin; KB: Klüver-Barrera; KO: knockout; PBS: phosphate-buffered saline; PD: Parkinson’s disease; RT-PCR: reverse transcription-polymerase chain reaction; TKO: triple knockout; TLC: thin-layer chromatography; TNF-α: tumor necrosis factor-alpha; WT: wild-type.

## Competing interests

The authors declare that they have no competing interests.

## Authors’ contributions

YOhm and KoF helped to prepare animals and perform experiments, to conceive and design the experimental plan, and to write the manuscript. YOhk and OT helped to prepare animals and perform experiments. KeF helped to prepare animals and perform experiments and to conceive and design the experimental plan. YS helped to conceive and design the experimental plan. All authors have read and approved the final version of the manuscript.

## Supplementary Material

Additional file 1: Table S1 Sequences of primers used for real-time RT-PCR. **Figure S1.** Expression levels of complement components were up-regulated in the spinal cord of DKO mice. **Figure S2.** Expression levels of complement components in the spinal cord were regulated in a distinct manner from that in the liver. Click here for file
