# Supplementary material for: Ganglioside deficiency causes inflammation and neurodegeneration via the activation of complement system in the spinal cord
Source: J Neuroinflammation. 2014 Mar 28;11:61. doi: 10.1186/1742-2094-11-61 (PMC3986855; doi:10.1186/1742-2094-11-61)
Supplement: Additional file 1: Table S1 — Sequences of primers used for real-time RT-PCR. Figure S1. Expression levels of complement components were up-regulated in the spinal cord of DKO mice. Figure S2. Expression levels of complement components in the spinal cord were regulated in a distinct manner from that in the liver. [file 1742-2094-11-61-S1.pdf]

Additional documents:

Table S1: Sequences of primers used for real time RT-PCR

Figure S1: Expression levels of complement components were up-regulated in the spinal cord of DKO mice

Figure S2: Expression levels of complement components in the spinal cord were regulated in a distinct manner from that in the liver

Table S1 : Sequences of primers used for real time RT-PCR

Gene	NCBI (accession No.)	sense	antisense	product size (bp)
C1qα	NM_007572	acaaggtcctcaccaaccag	aagatgctgtcggcttcagt	307
C1qβ	NM_007574	tggcactccagggattaaag	atagttctcgttcgcgttgg	301
C1qγ	NM_009777	agtgccgggcctctactact	cgggaaacagtaggaaacca	251
C2	NM_013484	atccgtcacaccatcatcct	ttctttccagtccacatcca	168
C3	NM_009778	aactgctggcctctggagta	gcatgattcctcgaggttgt	212
*C3**	NM_009778	aactgctggcctctggagta	ctgtgttgttcacgcagctt	213
C4	NM_009780	ttaagacccacgggctacac	tggcatgtggtgttcttcttcat	169
C5	NM_010406	gctcttgtggaaggagtgga	aagcctggtgtcagaaatgc	228
C6	NM_016704	ctcaaccacctctcccagaa	ccagcaacaacaaagcctct	286
C7	XM_356827	cctcccagagatgatggaaa	ctggcaacaggatctccaat	257
C8α	AK090245	actgggtagagggcttccat	tggtctgtctgcttgtccag	212
C8β	AK050313	cttctgtcaggttggcttcc	cagaccagtcagaccagcaa	310
C8γ	AK005962	cttctgtcaggttggcttcc	tggtggttgctgagactgac	156
C9	NM_013485	aaggtttcgctcaagaagca	tccacagtcgttgtcaccat	199
C3aR	NM_007577	tgacaggtcagctccttcct	cattaggaggctttccacca	266
C5aR	NM_007577	caagacgctcaaagtggtga	tatgatgctggggagagacc	244
IL-1α	NM_010554	cgaagctctccgtacattcc	gaatccaggggaaacactga	263
IL-1β	NM_008361	gcccatcctctgtgactcat	aggccacaggtattttgtcg	230
TNFα	NM_013693	ccacatctccctccagaaaa	agggtctgggccatagaact	259
mGAPDH	NM_008084	ggtgctgagtatgtcgtgga	ccttccacaatgccaaagtt	248

* Primers used for TKO mice.

Figure S1. Expression levels of complement components were up-regulated in the spinal cord of DKO mice

Expression levels of complement genes in the spinal cord of individual mice were analyzed by real time RT-PCR and presented after correction with the mouse *GAPDH* gene. (A) Expression of the *C4* gene was up-regulated in both male and female 28-week-old and 48-week-old spinal cord of DKO mice compared with WT mice. (B) Expression of *C3aR* was up-regulated in the male spinal cord of 28-week-old DKO mice and in both male and female spinal cord of 48-week-old DKO mice compared with WT mice. The number of mice examined was: 28-week-old male WT n=3, DKO n=3; 28-week-old female WT n=3, DKO n=6; 48-week-old male WT n=3, DKO n=3; 48-week-old female WT n=3, DKO n=5; data are presented as mean ±SD. *, $p<0.05$; **, $p<0.01$; ***, $p<0.001$.

Figure S2. Expression levels of complement components in the spinal cord were regulated in a distinct manner from that in the liver

To properly compare the expression levels of complement genes in the spinal cord and liver, graphs for the individual genes as shown in Fig. 3 were combined with appropriate scales.

Fig. S1

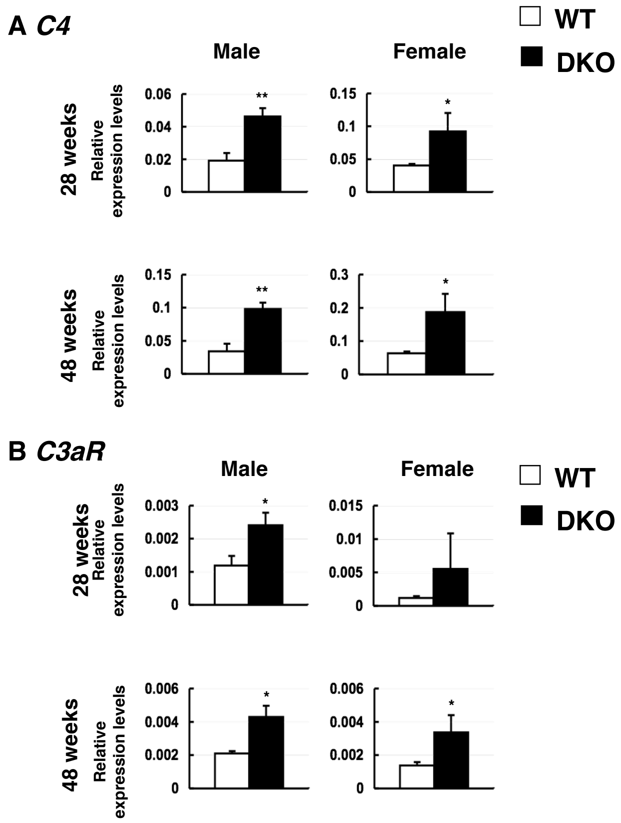
A C4
Male
Female
WT
DKO
28 weeks
48 weeks
Relative expression levels
0.06
0.04
0.02
0
0.15
0.1
0.05
0.3
0.2
**
*
B C3aR
0.003
0.002
0.001
0.015
0.01
0.005
0.006
0.004

Fig. S2

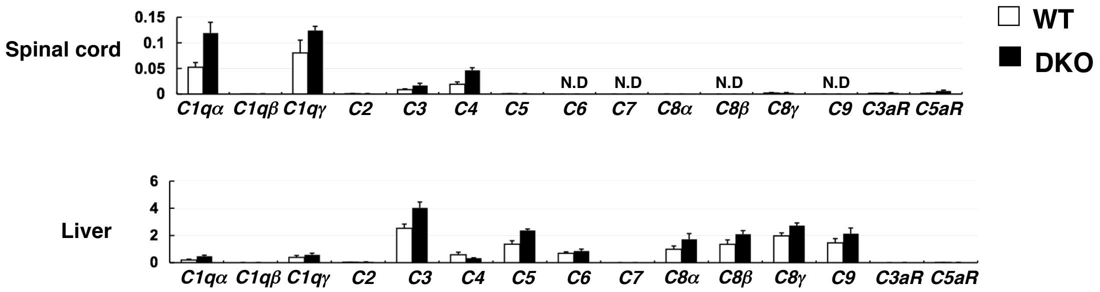
Spinal cord
0.15
0.1
0.05
0
N.D
N.D
N.D
N.D
C1qα C1qβ C1qγ C2 C3 C4 C5 C6 C7 C8α C8β C8γ C9 C3aR C5aR
WT
DKO
Liver
6
4
2
0
C1qα C1qβ C1qγ C2 C3 C4 C5 C6 C7 C8α C8β C8γ C9 C3aR C5aR
